# Which Is the Most Significant Cause of Aging?

**DOI:** 10.3390/antiox4040793

**Published:** 2015-12-17

**Authors:** Stefan I. Liochev

**Affiliations:** 8 Foxlair Court, Durham, NC 27712, USA; E-Mail: liochev@yahoo.com; Tel.: +1-919-382-8713

**Keywords:** aging, reactive oxygen species, synergy, theory, oxidative stress, free radicals, lifespan

## Abstract

It becomes clearer and clearer that aging is a result of a significant number of causes and it would seem that counteracting one or several of them should not make a significant difference. Taken at face value, this suggests, for example, that free radicals and reactive oxygen species do not play a significant role in aging and that the lifespan of organisms cannot be significantly extended. In this review, I point to the fact that the causes of aging synergize with each other and discuss the implications involved. One implication is that when two or more synergizing causes increase over time, the result of their action increases dramatically; I discuss a simple model demonstrating this. It is reasonable to conclude that this might explain the acceleration of aging and mortality with age. In this regard, the analysis of results and mortality patterns described in studies involving yeasts and *Drosophila* provides support for this view. Since the causes of aging are synergizing, it is also concluded that none of them is the major one but many including free radicals, *etc.* play significant roles. It follows that health/lifespan might be significantly extended if we eliminate or even attenuate the increase of a few or even just one of the causes of aging. While the synergism between the causes of aging is the main topic of this review, several related matters are briefly discussed as well.

## 1. Introduction

According to Harman [1], “Aging is the progressive accumulation of changes with time that are associated with or responsible for the ever-increasing susceptibility to disease and death which accompanies advancing age” and “the sum of the deleterious free radical reactions going on continuously throughout the cells and tissues constitutes the aging process or is a major contributor to it”. According to Hayflick [2], “The common denominator that underlies all modern theories of biological aging is change in molecular structure and, hence, function”.

While many authors believe that free radicals and oxidative stress play an insignificant role in aging (if any), it is hard to disagree with the clearly expressed view [2,3,4,5,6,7,8,9,10] that aging is the generated by multiple causes damage to the structures and functions of the molecules, cells, organs, *etc.*, of an organism. Such causes of aging include but are not limited to oxidative stress, glycation, telomere shortening, side reactions, mutations, aggregation of proteins, *etc.* In other words, it is the progressive damage to these structures and functions that we perceive and characterize as aging. This damage leads to development of pathological conditions and, as a consequence, to death. Hence, in agreement with the view expressed by Hayflick [3] and Holliday [4], we know the general cause of aging. This may be called the standard [10] or general (GTA) [11] theory of aging. Since it becomes more and more clear that aging is due to a significant number, or even a myriad, of causes [10,11,12,13], the term unified theory of aging is applicable as well. Those who disagree with it have to clearly define what else aging could be from a mechanistic point of view, regardless of their more philosophical views about why we age and why and how life, aging, and death came into being.

Opinions concerning the possibility of counteracting aging range from very optimistic to very pessimistic views and are described in numerous papers and comments on such papers. Therefore, only few recent papers that discuss such matters are cited here [14,15,16,17].

There is a view that lifespan can be extended dramatically and some even suggest that practical immortality is possible and even achievable in the foreseeable future. 

On the other hand, many are of the opinion that the human lifespan cannot be extended much beyond the current level, if at all, for the variety of reasons discussed in their papers. The view that there are myriad causes of aging, each one contributing insignificantly and thus too numerous to be protected against [12,13,18], seems especially pessimistic.

Yet there are numerous reports, only a tiny number of which can be cited here [19,20,21,22,23,24,25,26], that treatments, drugs, diets, phytochemicals, genetic manipulations of enzyme levels, *etc.*, significantly increase the lifespan and/or the health span of animals. While I presented arguments that free radicals, ROS, and oxidative stress play a significant role in aging [11,27], the important question that remains unsolved is how important that role is compared to the role of other often discussed and potentially significant causes of aging such as glycations, side reactions, enzyme infidelity, *etc.* Perhaps there are much more important causes of aging than free radicals and ROS? While I agree that there are many or even myriad causes of aging, I will nevertheless argue that many of them play significant roles and even appear as main causes.

Thus, there are numerous reports in the literature that the damage resulting from the action of a certain cause depends also on the action of other causes. In this regard, it is well known that in many cases agents of different types synergize in causing damage. Of importance is a hypothesis presented in a paper entitled “An emerging hypothesis: synergistic induction of aging by free radicals and Maillard reactions” [28]. It suggests that free radicals, glycation, and the Maillard reaction synergize in causing damage and in fact represent partially interactive elements of a single, more complex biochemical pathway. As noted by Kristal and Yu [28], such proposals might lead to a unified theory of aging. Another hypothesis suggests synergy between cancer and aging [29]. Thus, if the action of each of two or more causes is necessary for a substantial damage to occur, each of these causes is a significant one. 

Perhaps the most famous equation attempting to express in mathematical terms “the ever-increasing susceptibility to disease and death which accompanies advancing age” is the Gompertz Equation (1):

(1) *μ*(*x*) = *α*e^*βx*^,

where *μ*(*x*) is the mortality rate at age *x*, and *α* and *β* are constants [30]. 

The mortality and aging patterns of humans are comparatively well described by that equation [30,31].

This equation is sometimes called a “law” but exclusions apply [30,32]. The constants involved are not immutable, and unlike the physical constants change depending on the type of organism, as a result of the action of agents that cause or protect against damage, *etc.* [30]. For example, in the Mn SOD-deficient *Drosophila β* is higher than in the wild type [33]. Therefore, this equation is very useful but might be just a good approximation, while the nature of the underlying processes (it reflects) that increase the probability of death with advancing age is still debated. The hypotheses trying to explain these underlying processes and the interactions between them, among other things, include one based on the reliability theory [34] and the “network theory of aging” [35]. Several other equations including Weibull’s compete with Gompertz’s for better description of actual mortality patterns in humans and other organisms [30].

An interesting model of aging is based on the interaction between the process of generation of damage or, as the authors call it, “deficits accumulation,” and the decrease over time in damage control and recovery [36]. The existence of a “vicious circle” has been discussed recently [22,37]. Another interesting model suggests synergistic interactions between mutations, so that mutations that appear later have greater effects than those that appear initially [38]. It is important to note that any reasonable theory of aging (among the many existing) should be able to explain why the mortality rate increases and aging accelerates; theories that fail should be considered incorrect or in need of significant modifications. 

In this review, I discuss an explanation that follows similar logic and is based on kinetics and on the synergism and cooperation between the causes of damage and aging on one hand and of longevity and health assurance mechanisms on the other. The term “assurance mechanisms” describes enzymes, other agents, and systems involved in protection (such as superoxide dismutase) as well as in repair (such as proteasome) of the damage and is further defined in a couple of the cited reviews [10,11]. Hence, it is reasonable to suggest that significant damage (aging) occurs when the force (level) of more than one cause of aging increases substantially and/or if the efficacy of more than one assurance mechanism decreases considerably over time. In this case, if the causes of aging (structural and functional damage) interact synergistically in causing damage, the rate of generation of damage, and hence the rate of aging, increases dramatically with time. As a result, the failure of essential health and/or longevity assurance systems leads to the development of pathological conditions and finally to death. Early in life, the assurance systems function optimally and therefore the rate of aging is slow and almost flat. The rate of aging and thus mortality increases when the rate of damage generation increases and the assurance mechanisms start failing later in life. A recent review [39] provides an interesting discussion about the existence of latent and manifest (accelerating) stages of mortality and aging in humans and discusses many relevant papers. More discussions about these stages in models involving different organisms and the factors that modulate them are presented later in the text. 

Hence, in principle, a substantial increase in health and lifespan might be achieved if we manage to substantially restore the function of one or more failed assurance mechanisms. This view is certainly more optimistic than the absolutely pessimistic one, but could be called mildly skeptical since it posits that a significant or at least moderate but not unlimited increase in health and lifespan might be achievable in the foreseeable future.

Thus, the significance of the synergism between the causes of damage and aging is the main focus of this review but some related matters such as the significance of free radicals and oxidative stress as causes of aging are discussed as well.

## 2. Lessons from a Simple Model

One of the first mechanisms proposed in order to explain the toxicity of (ROS) is based on the formation of the highly reactive and mutagenic hydroxyl radical (•OH), or an equally reactive intermediate, by the Fenton reaction [40,41,42,43,44]: Fe (II) + H_2_O_2_ → Fe (III) + •OH + HO^−^. Here Fe (II) and Fe (III) denote the so-called “free iron” complexes.

The rate equation of this reaction Equation (2) is:

(2) v = k [H_2_O_2_] [Fe (II)],

where k is the second order rate constant.

Let us now make several more or less reasonable proposals. Imagine that the Fenton reaction is the only mechanism that causes aging-related damage in certain hypothetical organism(s). Let us propose also that the concentrations of both H_2_O_2_ and Fe (II) increase (due to aging-related changes) in proportion with their initial value over time, according to Equation (3), which allows the calculation of the rate of the Fenton reaction at given time:

(3) v_t_ = k (*n* + 1) [H_2_O_2_]_0_ (*n* + 1)[Fe (II)]_0_ = v_0_ (*n* + 1)^2^

In this equation, *n* is a number that characterizes the increase of the concentrations of the reactants and of v_0_ per time period, v_t_ is the rate at a given time, v_0_ is the initial rate before the concentrations of the reagents start to increase, and [H_2_O_2_]_0_ and [Fe (II)]_0_ are the concentrations at time zero. For simplicity, n is taken to be the same for both reactants.

In the case of many time periods, the equation becomes v_t_ = v_0_ (*xn* + 1)^2^, where *x* is the number of years or other time periods. For simplicity and for the purpose of demonstrating the power of synergy in an uncomplicated way, suppose that n is equal to 1 per year or per certain time period. 

Thus, if *x* is the number of years, after one year the concentrations of Fe (II) and of H_2_O_2_ will be twice as large as at time zero, while the rate of production of •OH and hence the rate of the damage being inflicted by it will be four times higher. After two years the corresponding numbers will be 3, 3, and 9; after three years 4, 4, and 16, and so on.

Suppose now that the level of one of the reactants does not increase with time, perhaps because the organisms have been treated by a perfect catalase mimic or a chelator for Fe (II). In this case, if Fe (II) is the reactant whose concentration does not increase with time, Equation (3) reduces to Equation (4):

(4) v_t_ = k (*n* + 1)[H_2_O_2_]_0_ [Fe (II)]_0_= v_0_ (*n* + 1)

In this situation, after one year the concentration of H_2_O_2_ doubles, the concentration of Fe (II) remains the same, and the rate doubles. After two years [H_2_O_2_] triples, Fe (II) remains unchanged, v triples, and so on.

**Figure 1:**
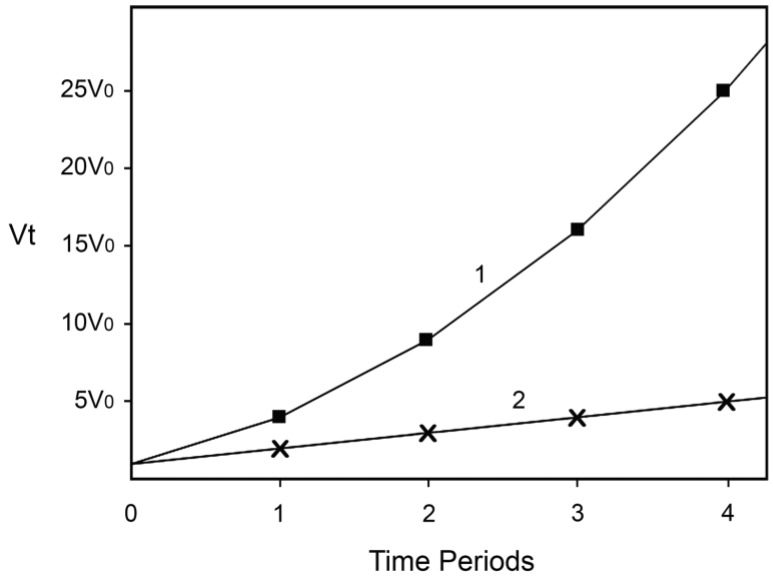
Increase of the rate of the Fenton reaction due to increase of the levels of the reactants. Line 1: The levels of both reactants increase. Line 2: The level of only one of the reactants increases. The details and the assumptions are described in the text.

Finally, let us suggest that when the rate of the Fenton reaction increases 16-fold, the damage inflicted causes directly or indirectly the death of 50% of the hypothetical organisms, perhaps because they cannot tolerate and repair so much damage being inflicted. In the case described by Equation (3) this will happen after three years, while in the case described by Equation (4) after 15 years. If for (hypothetical) humans x is the number of 20-year long periods, this means that without preventing one of the causes to increase, 50% will be dead after 60 years, while if we succeed in preventing the increase of one of the causes 50% will be dead after 300 years! Not as good a result as if we could prevent the increase of both causes, but still an impressive one. Note also that very little time is needed for the rate of the Fenton reaction to increase from a level at which most hypothetical organisms will be able to tolerate and repair the damage generated (somehow less than 16-fold increase) to a level that will kill most of them (slightly more than 16-fold). Hence, the model predicts “ever-increasing susceptibility to disease and death which accompanies advancing age” and dramatic increase of aging and mortality later in life when the rate of damage generation overwhelms the assurance mechanisms that counteract the damage. The dependence of v_t_ on time is represented on Figure 1. Equation (3) is represented by line 1 and Equation (4) by line 2.

The reality is much more complex than this simple model. Certainly there are more than just two synergizing causes. A better model of aging should also consider that the failure of one assurance mechanism is likely to cause damage to other assurance systems and that some of the interactions between the causes might be partially synergistic, as suggested by Kristal and Yu [28]. Finally, the simple model predicts the acceleration of aging by the interaction of certain causes but falls short of predicting the acceleration of mortality, which is better described by equations such as Gompertz’s. In this regard it should be kept in mind that the acceleration of mortality is due to a complex interaction of intrinsic and extrinsic causes [45]. Hence, a more general equation describing the totality of interactions will be more appropriate. Hopefully, readers more experienced in mathematical modeling might become interested enough to elaborate.

In this model, the increase of the levels of the substrates of the Fenton reaction or of the failure of the repair systems might be lower or higher and n may be different for different causes. Yet, as much as the synergizing causes of aging keep increasing, the rate of damage will be accelerating dramatically. 

It should not be assumed that the reactants of any second-order reaction necessarily synergize in causing damage. In this regard, the hypothetical case analyzed above is not just based on assumptions. The free (non-heme) iron level increases during aging and an iron chelator increases both the lifespan and the health span of *C. elegans*, while decreasing protein aggregation [46,47,48]. The levels of ROS, including the level of H_2_O_2_, generally increase during oxidative stress and aging as a result of increased production and/or of decreased activity or concentration of the enzymes and antioxidants that scavenge them [22,37,48,49]. The increase of the individual ROS and other potential causes of aging is sometimes small [22]. However, an important feature of the proposed model is that relatively small increases in the individual cooperating causes of damage lead to a dramatic increase in the rate of damage and thus the probability of dying,

The analysis in this section is made for the purpose of illustrating the significance of the synergism between the causes of aging (damage) and not for suggesting that the Fenton reaction is the major cause of aging. In reality, there are many aspects to consider and we should not realistically expect such dramatic results by preventing the increase of just one cause of damage. For example, in the above case, the hypothetical mimics or chelating agents must be so perfect that they prevent the increase of the reactants but do not decrease them too much. This is the case since both H_2_O_2_ and iron, while participating in reactions that generate toxic intermediates, are, nevertheless, biologically important metabolites. In this regard, the toxic Fenton reaction proceeds even at normal conditions and the damage caused by it is inevitable. In fact, one of the main reasons immortality and even dramatic increase of longevity and of health span are impossible is that the very agents, such as sugars, H_2_O_2_, iron, oxygen, sunlight, *etc.*, that we need for survival are, on the other hand, even at normal levels, more or less direct causes of aging and thus of our demise. Hence, “the sum of the deleterious free radical” and other “reactions going on continuously throughout the cells and tissues” cannot become zero. In other words v_0_ cannot become zero.

## 3. The Phenomenon of Synergism

Synergy (synergism) is usually defined as “the interaction of two or more agents or forces so that their combined effect is greater than the sum of their individual effects”. When one starts to think about it, one finds that synergism is everywhere. Thus, bacteria and viruses synergize in causing infection, other causes of diseases synergize, drugs synergize, *etc.* Here, I will comment only on two types (cases) of synergism. In the first case, causes of a certain type of stress synergize in causing damage and in the second one different stresses synergize in doing so.

Soon after the discovery of the biological function of superoxide dismutase (SOD), it was found that superoxide (O_2_^•−^) and H_2_O_2_ synergize in causing toxicity. First it was thought that they react directly in the so-called Haber–Weiss reaction to produce •OH, which is a very reactive species. However, this reaction does not occur directly and it was found that for synergism to occur reactive iron was also required. This led to the hypothesis that O_2_^•−^ reduces Fe (III) to Fe (II), which in turn reduces H_2_O_2_ in the Fenton reaction producing •OH and Fe (III). This toxic pathway may occur in some situations but what happens intracellularly is different. In that case, O_2_^•−^ oxidizes iron-containing enzymes, which leads to the release of Fe (II) followed by the Fenton reaction. Finally, cellular reductants reduce Fe (III) back to Fe (II). All of this has been discussed [43,44] and it should be noted that establishing the mechanism of a certain synergy is usually a difficult task. 

Interestingly, unlike in the case of other species, the SOD-null mutant of *C. elegans* has the same lifespan as the wild type, despite being obviously sick and displaying typical aging characteristics [50]. It was also much more sensitive than the wild type (in the sense of being killed) towards various stresses such as cold stress and osmotic stress. In this case, it might be said as well (having in mind the definition of synergy) that a given stress, for example cold shock, makes the animal more sensitive to the oxidative stress due to the lack of SOD. Clearly synergy is involved but the mechanism of the synergy between the causes of cold shock and these of oxidative stress is not yet clear.

Finally, old people are much more sensitive to various diseases and stresses. It seems reasonable to conclude that in this case causes of aging and other exogenous and endogenous causes of damage are synergizing as well. The interaction between such extrinsic and intrinsic causes and the importance of these interactions in mortality acceleration has been discussed in detail very recently [45].

The important point in all these cases is that eliminating just one cause or type of causes eliminates the synergy and in this way the worst consequences of that synergy.

## 4. Analysis of Some Cases from the Theory and Practice of the Science of Aging

**Case 1.** Paul *et al.* [33] found that Mn SOD-deficient *Drosophila* has a lower lifespan than the wild type and provided evidence that this mutant displays characteristics similar to these of aging. This has been reported for many other animals and for other SODs. The health span of SOD-deficient animals is also affected. Most recently, Ivannikov and Van Remmen [51] found that the deficiency of Cu, Zn, and SOD in adult mice causes specific kinds of damage that also occur in old wild-type mice and suggested that these changes in the old wild-type mice might in part be due to reactive oxygen species (ROS).

Importantly, the analysis of the experiments of Paul *et al.* [33] provides evidence that a significant increase in mortality occurs when the rate of generation of damage increases substantially. These authors have created a number of *Drosophila* mutants having progressively lower levels of MnSOD, which resulted in progressively shorter lifespans. What is most interesting, however, is that in the essentially flat lag period of the first 10 to 20 days, the mortality of the wild-type animals was not different from that of the mutants and therefore the initial mortality rate (*α*) was not influenced by the SOD-deficiency. The author’s analysis, based on the Gompertz model, confirmed that. In the same time, *β* as well as *μ* increased in proportion with the extent of the SOD deficiency. What could explain such behavior? The apparent explanation, according to the model discussed above, is that the increased level of O_2_^−^ became more toxic only after other causes of aging (damage) increased in time, likely due to a failure of some other assurance mechanisms. In that situation, the increased O_2_^•−^ level synergized with these other causes of damage (aging), thus accelerating the rate of aging and promoting death. As discussed in Section 3 and earlier [27], superoxide needs the cooperation of other species in order to kill. 

Resveratrol significantly extended the lifespan of *C. elegans* and exerted other beneficial effects, while the negative tradeoffs were minimal [52]. Interestingly, resveratrol increased the lifespan by extending the lag phase, while the exponential phase was not affected. This indicates that resveratrol did not act by directly scavenging free radicals, ROS, or other reactive species, since such action should have decreased *β*. It was pointed out, in this regard, that the beneficial effects of phytochemicals and other agents and specifically of resveratrol cannot be due to direct scavenging of reactive species, since their intracellular concentrations are low and thus they cannot efficiently compete with numerous abundant intracellular targets for the reactive species [52,53]. The beneficial effect of phytochemicals is thought to be due to hormesis [54]. Therefore, it is tempting to speculate that resveratrol caused beneficial adaptation(s) that delayed the failure of essential assurance system(s) and thus the synergy between important causes of aging. In fact, the beneficial effect of resveratrol in several organisms including *C. elegans* and mice is likely mediated by sirtuins and by peroxisome proliferator-activated receptor-γ coactivator 1α (PGC-1α) and subsequently affects mitochondrial function [55].

It seems that the low initial mortality rate, which was not affected by O_2_^•−^, reflects a state where the assurance systems function with maximal efficiency and the organism is able to adapt [33]; there is little synergy between the causes of aging whose intensity in that period is also low. Within the framework of the Gompertz model , this supports the opinion [56] that *α* reflects the basic protection of a given system against failure and damage, while *β* indicates the rate of deterioration of that protection. According to Kowald [56], the Gompertz equation reflects aging and hence *μ*(*x*) represents aging and not just mortality. 

The parameters *α* and *β* are more or less analogical to v_0_ and n, respectively, in the model described above. Hence, it might be concluded that to a significant extent we age and the rate of aging is increasing since the rate of the aging-related damage generation (v_t_) is increasing faster and faster with the passage of time due to the increasing power of the synergizing destructive forces (causes of aging). Species and individuals that have low v_0_ and n might be expected to have long lifespans. Certainly the abilities of the organisms to tolerate and repair the damage being inflicted, as well as the rate of decline of these abilities with time are also important longevity-determining factors. Note that the failure of systems that repair the damage will increase the recovery time, which is an important factor in the model of Mitnitski *et al.* [36]. Hence, both the increase of the rate of damage generation and the increase of the recovery time are factors that determine the rate of aging. 

**Case 2.** The evolutionary theories of aging have been reviewed and there is a view that while the mechanistic theories of aging attempt to answer how we age, the evolutionary theories can explain why we age [57,58]. It should be kept in mind that, according to Gavrilov and Gavrilova [57], these theories might not be yet “ultimate completed theories, but rather a set of ideas that themselves require further elaboration and validation”; Le Bourg [58] expresses similar thoughts.

According to some such theories, we accumulate negative traits (like those causing some inheritable diseases) when they negatively affect us after reproduction but not earlier, since such traits cannot be eliminated by selection. First, this is not entirely so, at least in the case of humans and other organisms where wisdom (fitness) of the aged contributes to the survival of the young. Hence, if a care-giving parent has such a trait, the progeny is at a disadvantage and will be subjected to increased natural selection pressure. What is more relevant to the present discussion is that the evolutionary hypotheses do not quite explain from a mechanistic point of view why such diseases start after reproduction. What is the clock? The apparent explanation is that in these cases there is actually a synergism between two kinds of processes. One of them is aging, which is the unavoidable damage to essentially all functions and structures, and the second is the toxicity due to the inherited negative trait. This synergism is likely to start after a lag, because the assurance mechanisms that are important in counteracting the toxicity caused by the negative trait are still largely intact initially until they start to fail at a later time.

Thus, evolutionary theories alone do not explain important aspects of why we age. Aging is an unavoidable process of deterioration that can be modulated but not completely stopped by evolution; sooner or later it will cause the development of pathological conditions and death—this is why we age. True, evolution may increase the lifespan significantly in a number of ways including by switching the balance between longevity/health assurance mechanisms and reproduction in favor of the former [6,58]. However, due to the limited resources available, the creativity of evolution cannot prevent the final result of the relentless action of the destructive forces. Therefore, the questions of why and how we age should be answered mostly by mechanistic theories. Evolutionary theories, however, should be able to better and better explain how and why aging is modulated (accelerated or slowed down) by evolution. Thus Kirkwood and Melov [59] discuss the role of natural selection in “shaping” ageing. Such knowledge will certainly help us to design better strategies to “fight” aging. In this regard, what is the evolutionary explanation for cases where fertility decreases before death? The beginning of the answer, which is important for the discussion later in the text, might be that in this way the birth of significantly defective individuals by parents who acquire inheritable defects due to age-related damage *during their individual lifetime* is avoided. Certainly, as in the case of humans, there are other reasons as well [60].

This being said, there should be at least two ways to counteract diseases that develop after reproduction. One is to repair or prevent the damage caused by the toxic mechanism triggered by the disease and the second is to prevent the failure of, or to repair, the affected through aging-relevant assurance mechanisms. 

Of course, it is diabolical that the causes of aging and/or diseases synergize and accelerate each other’s development. On the other hand, once we learn how they interact, it is easier to find at least a partial treatment by counteracting just some or even one of the interacting causes. 

A more specific discussion about some of the points raised follows.

**Case 3.** Damage to DNA and increased mutation accumulation are often suggested as significant causes of aging and results of aging [38,61,62,63,64]. Interestingly, in a recent study involving yeasts, Kaya *et al.* [65] assessed the mutations appearing in colonies of daughter cells of young and old mother cells, respectively, and found that although the number of mutations increased with age, the numbers were surprisingly low. They estimated less than one mutation per (replicative) lifespan, although this might be an underestimation, as they noted. They also did not observe significant structural genomic changes in those cells.

Kaya *et al.* [65] interpreted these results to mean that mutations and genomic changes in general do not play a significant role in the aging of yeasts and at face value one should conclude that they are not a significant result of aging either. According to them, mutations belong to a category of myriad “mild damage forms…which taken in isolation do not cause aging, aging may still result from cumulative damage, to which these damage forms contribute”. Finally, according to them, “the mild damage forms are too numerous to be protected against”, which is in agreement with the previously expressed views of one of the coauthors, who declared the free radical theory of aging (FRTA) dead as well [13].

Taken at face value and especially if valid for other organisms and not just for yeasts, these conclusions suggest that many concepts and theories about the role of oxidative stress and of mutations in aging are likely wrong or badly in need of significant modifications. Moreover, free radicals and oxidative stress, which are considered to be important causes of aging [1,11,27], cause damage to DNA and mutations, as discussed above and later in the text. Finally, defective genome maintenance and DNA repair are known to promote phenotypes of premature aging [66]. It is worthwhile, therefore, before committing such concepts to the garbage bin of history, to critically analyze the work of the authors and some related studies in order to better understand the process of aging in general and specifically in yeasts.

Several papers cited by Kaya *et al.* [65] demonstrate that during their replicative lifespan yeasts accumulate some damage and age [67,68,69,70]. Thus, old cells pass some of the damaged molecules, aggregated proteins, and dysfunctional mitochondria to their daughter cells and daughters of very old mothers live a shorter (replicative) life. Interestingly, granddaughters of old mother cells are apparently able to clear the damage and their lifespan is restored to that of daughters of young mothers.

It should be concluded that while yeasts age and accumulate some damage during their replicative lifespan, neither the rate of aging nor the damage accumulated was extensive enough to permanently damage even the last daughter of a mother; indeed such daughters produced colonies, as noted by Kaya *et al.* [65]. What was just discussed is important for several reasons and one concerns the validity of the conclusions of Kaya *et al.*

Kaya *et al.* [65] discussed an apparently contradictory study. Thus, Hu *et al.* [71] found significant numbers of varieties of genomic changes in populations of very old cells, most of which have ceased to divide. As Kaya *et al.* [65] noted, it is likely that these changes occurred after the last daughter was budded. They further speculated that these changes might have contributed to the death of the old cells, defining death as an inability to bud one more daughter, but not to the process of aging *per se*, although this does not explain what caused these changes. However, in reality, the yeasts that have just ceased to divide are just as “dead” as other early postmitotic and senescent cells and as women in an early post-menopausal period. In fact, all of them live for more time after that and continue to be subject to aging. The destructive forces never rest. That senescent cells do not die immediately after their last daughter is budded and remain metabolically active for days after that is known [68]. In this regard, Zadrag-Tecza *et al.* [72] and Minois *et al.* [73] have warned that cells that have just ceased to bud daughters are not yet dead.

Therefore, it might be more reasonable to suggest that genomic changes and other forms of damage result from aging and play a significant role in the aging that occurs in a mother *after* the last daughter is budded but not before that moment. Indeed, as stated by Vijg [64], it is likely that mutations accumulate more readily in postmitotic tissues (cells) than in actively proliferating ones. Furthermore, it is likely that aging is comparatively moderate until or just before the last daughter is born but accelerates after that. To learn more about aging as it continues in the cells that have ceased to divide and/or organisms in post-reproductive periods and in the last stages of life, we have to analyze several more papers.

Laun *et al.* [74] present evidence for a significant oxidative stress in old (senescent or nearly senescent) but not in young yeast cells.

A more recent paper by Brandes *et al.* [75] is also informative, although they studied chronologically aging yeasts that stopped dividing when they approached the stationary phase. They observed that soon after the cells stop dividing a pro-oxidizing shift occurs. Specifically, the level of NADPH decreases, while protein thiols become dramatically oxidized. In some cases the oxidation was auto-accelerating. It is also evident from their results that, at the same time or shortly after these events, the mortality of the yeasts, which was negligible initially, started to accelerate. It might be concluded that after the yeasts ceased to reproduce, they experienced accelerated aging and damage, suggesting synergizing causes in action. This accelerated aging led to pathological conditions and to real (final) death and not just the reproductive one of the yeasts. 

Here is a summary of what we have learned so far as a result of the analysis made: During the replicative lifespan of yeasts some damage, such as protein aggregates and some mutations, accumulates and this potentially may lead to further damage. The damage accumulated during the replicative lifespan, but not due to mutations, is sufficient to eliminate the ability of the cells to reproduce but does not kill them in the literal sense of the word. In the subsequent period, as a result of further damage, aging accelerates, likely because the causes of aging, whose levels increase, synergize with each other and this finally leads to death. The synergizing causes likely include genomic changes and aggregates formation, as well as other forms of damage such as oxidative stress. 

The end of the replicative lifespan of yeasts is the end of their replicative period and not of their life [72], and the results of Kaya *et al.* [65] strongly suggest that mutations are not part of the mechanism that stops the replication. The nature of this mechanism has been discussed recently by Bilinski and Zadrag-Tecza [76]. In this regard, oxidative stress is a good candidate for a mechanism that stops replication or might at least be one of the factors that triggers it. Thus, SOD or glutathione peroxidase-deficient mutants have dramatically shorter replicative lifespans [72,77]. It is likely that evolution preserved this mechanism and may have even perfected and synchronized it, since production of significantly impaired daughters is unlikely to be beneficial for the group and for the species. The analogy with humans is evident. Bilinski and Zadrag-Tecza [76] urge caution about the use of yeast as a model organism of gerontology. Anyway, it seems obvious that correct understanding about the nature of aging in yeasts as well as in humans cannot be achieved by studying only the changes that occur prior to the end of the reproductive lifespan. Thus, evidently, yeasts and many other cells and organisms might be considered to be in the lag phase of aging or in the early period of acceleration, where aging, although progressing, has not accelerated significantly, at least until approximately the last phase of the reproductive period. More about the timeline of the switch from the lag period, which also might be called the latent mortality period, to that of accelerated aging and mortality in humans can be read in the review by Salinary and De Santis [39]. All of this should not be taken to mean that aging-related damage (including mutations) does not occur at all in dividing cells and prior to the end of reproduction and that such damage cannot contribute to the development of diseases, especially in organisms having longer chronological lifespans than yeasts. 

One might speculate that the pro-oxidizing shift observed by Brandes *et al.* [75] is species dependent or condition dependent. As the authors discuss, this is not likely since similar changes were observed in experiments with aging rodents as well. Some of the most definitive results have been obtained in the laboratories of Sohal and Orr, who observed a similar pro-oxidizing shift in aging fruit flies [78,79]. Furthermore, they made a significant number of important observations such as that overproduction of antioxidant enzymes such as glucose-6-dehydrogenase, thioredoxin reductase, and peroxiredoxins 3 and 5 increased the lifespan of *Drosophila* and that the lifespan of *Drosophila* underexpressing both peroxiredoxin 3 and peroxiredoxin 5 was five times shorter than that of the wild type [69]. The significance of the findings of Sohal, Orr, and coworkers was underscored in my previous review [11]. 

The results of Brandes *et al.* [75] and of Sohal, Orr, and coworkers support FRTA despite the lack of clear evidence that the production of ROS was increased substantially. What these authors have observed in aging yeasts and *Drosophila* is the dramatic development of oxidative stress, since the state reached after the pro-oxidizing shift is oxidative stress and should be called so. According to the original and still valid definition of Sies [80], oxidative stress is “a disturbance in the pro-oxidant–antioxidant balance in favor of the former.” In other words, oxidative stress may result either from increased production of free radicals, ROS, and other oxidants or from a decreased ability of enzymes and antioxidants to scavenge these species and/or repair the damage caused by them. Note that in all of these cases, the species that cause the damage are the free radicals, ROS, and other oxidants; this is the essence of FRTA/oxidative stress theory of aging! In this regard NADPH and thiols are important for the reductive repair of inactivated enzymes, oxidized forms of antioxidants, *etc.*, among other things. This topic was discussed previously as well [11]. However, free radicals and even oxidants in general, although important causes of aging, are only one of the causes of damage and thus of aging. This was realized by many [1,9,10,12,13,28] and hence FRTA is only a part, though an an important one, of GTA [11].

Very important for the topics under discussion are observations made by Gladyshev and colleagues. They prepared and analyzed a mutant yeast strain (designated Δ8) lacking eight thiol peroxidases (peroxiredoxins and glutathione peroxidases) involved in the elimination of H_2_O_2_ [65,77,81]. This strain had a significantly shorter *replicative* lifespan and obviously accumulated serious damage, leading to a dramatic increase in point mutations and decreased growth rate as well. Furthermore, when the mutant was subjected to long-term mutation accumulation, the lifespan and the growth rate were further reduced. All of this seems to suggest that mutations that eliminate or decrease the activity of certain enzymes are capable of causing aging, in this case mostly by causing oxidative stress, which in turn resulted in many more mutations. Obviously, the damage caused by the cooperative actions of these mutations and other causes was significant enough to result in early aging and dramatically affect even the reproductive ability of the mutant. Furthermore, it might be expected that aging will accelerate faster under stresses, which the wild type will inevitably experience. Remarkably, expression of even a single thiol peroxidase could significantly attenuate the phenotype of ∆8 cells [77].

An observation of Fomenko *et al.* [81] is important for the present discussion and for the discussion [11] about the role of H_2_O_2_ and other ROS as signaling and/or toxic and aging-causing agents. Thus they found that the Δ8 mutant was unable to activate and repress gene expression in response to H_2_O_2_. As discussed by the authors, this suggests that H_2_O_2_ is involved in the process of redox signaling only because it is a substrate of the peroxidases, while side reactions with other thiols, proteins, *etc.* play an insignificant role, if any, in contrast with what many believe. It seems logical to conclude that these side reactions of H_2_O_2_ cause damage, which needs to be constantly (reductively or otherwise) repaired, rather than play a role in specific signaling. 

While the analysis of the relevant literature might be continued practically forever, the one made so far seems to essentially strongly support if not prove the points of discussion.

## 5. Conclusions

Due to the existence of synergistic interactions between the causes of aging, our perception of which causes are most important is influenced by a relativistic effect. Thus, to the observers investigating the toxicity of free radicals and/or their role in aging, they appear as the main cause, while the other causes appear insignificant, but the opposite appears to be the case for those that study other causes. Both are correct but also incorrect, specifically since the “other causes” are not insignificant! In reality, the processes are interdependent and it cannot be said that a given cause is responsible for 3% of aging, while another one for 33.3%. This undermines the arguments of those who suggest that FRTA is dead or about to die, while certainly the free radicals and ROS are not the only important causes of aging. The same could be said for other theories, so such theories are still alive, at least for now.

An important thesis, shortly described here, is that species longevity has been determined and conserved by millions of years of evolution and hence cannot be significantly improved, whatever we do. It is true that millions of years of evolution have resulted in longevity and health assurance systems of amazing complexity and efficacy. Although these systems cannot be perfect [11], it is extremely unlikely that in the foreseeable future we will be able to significantly improve such systems when their performance is at its peak. However, due to the inevitable damage caused by the forces of destruction that are the cause of aging, the efficacy of these systems progressively decreases. We are beginning to understand why and how this happens. Hence, prevention and/or repair are possible and likely to significantly extend both health and lifespan, and it is not clear what theoretical reasons might prove the opposite.

I do not share the view that near-immortality is possible, for reasons only inferred above; in the cause of brevity they cannot be discussed in more detail here. I also agree with the view [14,17] that the increase of lifespan will eventually slow down and approach a limit (the question is: to what extent could the maximal lifespan be increased by novel approaches?). In this regard, the view that by prevention and/or repair health and lifespan can be at least moderately extended seems logical to me. The mere fact that a variety of treatments and agents extend them in practice means that the theory that this cannot be done is incorrect and needs to be modified. In fact, a theory based on synergistic interactions between the causes of aging provides a plausible explanation for the beneficial effect of even simple treatments. Therefore, the problem is whether health and lifespan can be extended but how to achieve it. The strategy of taking excessive amounts of antioxidant vitamins has been proved inefficient; I have discussed why previously [11,27,53]. We take essential vitamins and minerals from the environment and we do not need more than a certain level of them. However, many people in the world still have an inadequate diet and for them taking supplements will be useful.

However, the case when certain assurance systems fail as a result of aging is different. In such cases prevention and/or repair of the damage to these systems should be useful. For example, proteasome and methionine sulfoxide reductases are damaged during aging and treatments that increase their activity or expression increase lifespan, while the underexpression of these enzymes decreases lifespan and the resistance to oxidative stress in a number of models [24,82,83]. Another situation I discussed recently [11] is that when an enzyme is increased during aging and this is toxic, treatments that decrease its activity or expression might be beneficial. This does not preclude the possible scenarios. Using treatments such as perfect mimics or genetic manipulations for definitively correcting such problems is an option but hormesis is another option. Hormesis is based on adaptation and adaptations are tradeoffs [11,84]. This does not mean that treatments based on hormesis cannot be very useful in many situations, as correctly argued recently by Le Bourg and Rattan [85]. Suppose that in certain species or individuals one assurance system is of excellent quality, while another one is shoddy and likely to fail early and thus determines longevity. It should be expected that improvement of the latter at some expense of the former will be beneficial.
